# Bond Strength of Adhesive Systems to Calcium Silicate-Based Materials: A Systematic Review and Meta-Analysis of In Vitro Studies

**DOI:** 10.3390/gels8050311

**Published:** 2022-05-18

**Authors:** Louis Hardan, Davide Mancino, Rim Bourgi, Alejandra Alvarado-Orozco, Laura Emma Rodríguez-Vilchis, Abigailt Flores-Ledesma, Carlos Enrique Cuevas-Suárez, Monika Lukomska-Szymanska, Ammar Eid, Maya-Line Danhache, Maryline Minoux, Youssef Haïkel, Naji Kharouf

**Affiliations:** 1 Department of Restorative Dentistry, School of Dentistry, Saint-Joseph University, Beirut 1107 2180, Lebanon; louis.hardan@usj.edu.lb (L.H.); rim.bourgi@net.usj.edu.lb (R.B.); 2 Department of Biomaterials and Bioengineering, INSERM UMR_S 1121, Biomaterials and Bioengineering, 67000 Strasbourg, France; endodontiefrancaise@outlook.com (D.M.); youssef.haikel@unistra.fr (Y.H.); 3 Department of Endodontics, Faculty of Dental Medicine, Strasbourg University, 67000 Strasbourg, France; m.minoux@unistra.fr; 4 Centro de Investigación y Estudios Avanzados en Odontología, Facultad de Odontología, Universidad Autónoma del Estado de México, Jesús Carranza esq. Paseo Tollocan, Col. Universidad, Toluca 50130, Mexico; alejandra.alv.orz@gmail.com (A.A.-O.); lerodriguezv@uaemex.mx (L.E.R.-V.); 5 Facultad de Estomatología, Benemerita Universidad Autónoma de Puebla, 31 Poniente #1304, Colonia Volcanes, Puebla 72410, Mexico; abigailt.flores@correo.buap.mx; 6 Dental Materials Laboratory, Academic Area of Dentistry, Autonomous University of Hidalgo State, Circuito Ex Hacienda La Concepción S/N, San Agustín Tlaxiaca 42160, Mexico; 7 Department of General Dentistry, Medical University of Lodz, 251 Pomorska St., 92-213 Lodz, Poland; monika.lukomska-szymanska@umed.lodz.pl; 8 Department of Endodontics, Faculty of Dental Medicine, Damascus University, Damascus 0100, Syria; ammarendo89@gmail.com; 9 Department of Stomatology, Robert-Debré Hospital, 75019 Paris, France; mdanhache28@gmail.com; 10 Friedrich Miescher Institute for Biomedical Research, Maulbeerstrasse 66, 4058 Basel, Switzerland

**Keywords:** adhesive systems, bond strength, calcium silicate-based cement, composite

## Abstract

Since the adhesion of resin composites to calcium silicate-based cement is considered challenging. Therefore, the best adhesion strategy should be indicated. This review aimed to assess the effect of different adhesive systems on the bond strength of resin composite to calcium silicate-based cement through a systematic review and meta-analysis. The subsequent PICOS framework used was: population, calcium silicate-based cement; intervention, use of self-etch adhesive systems; control, use of total-etch adhesive systems; outcome, bond strength; study design, in vitro studies. The literature search was conducted independently by two reviewers up to 18 February 2021. Electronic databases (PubMed, ISI Web of Science, SciELO, Scopus, and Embase) were searched for applicable articles. In vitro manuscripts studying the effect of adhesive systems on the bond strength of calcium silicate-based cement were considered. The meta-analyses were performed using Review Manager Software version 5.3.5 (The Nordic Cochrane Centre, The Cochrane Collaboration, Copenhagen, Denmark). Bond strength comparisons were made considering the type of calcium silicate-based cement (Mineral Trioxide Aggregate (MTA), Biodentine™, or TheraCal LC^®^). A *p*-value < 0.05 was considered statistically significant. A total of 7321 studies were retrieved in databases searched. After full-text evaluation, 37 eligible papers were assessed for qualitative analysis, leaving a total of 22 papers for the quantitative analysis. According to the meta-analysis, the bond strength values of resin composite materials to MTA and TheraCal LC^®^ cement were favored when a total-etch adhesive system was used (*p* ≤ 0.02). On the other hand, the meta-analysis of the bond strength of resin-based materials to Biodentine™ calcium silicate-based cement was similar between both approaches (*p* = 0.12). The in vitro evidence suggests that the bond strength of resin-based materials to both MTA and TheraCal LC^®^ cement was preferred by using the total-etch adhesive strategy. However, when bonding to Biodentine™, the use of self-etch or total-etch strategies displayed promising results. Given the lack of evidence related to the chemical interaction of self-etch adhesive materials with the bioceramics, if self-etch adhesives are used for bonding resin-based restorations to calcium silicate-based cement, a pretreatment with phosphoric acid could be recommended.

## 1. Introduction

Dental caries, restorative procedures, and tooth fractures may lead to pulp exposure and can endanger treatment prognosis [1]. When pulp exposure occurs, with the aim of maintaining pulp vitality, a biomaterial could be directly placed over the exposed pulp site (a clinical procedure called direct pulp capping) [2]. Other vital therapy procedures include indirect pulp capping (bioactive materials used as liners) and pulpotomy procedures (biomaterial applied following partial amputation of the dental pulp) [3].

A variety of pulp capping agents with specific properties, advantages, and drawbacks are available nowadays [4]. Novel biomaterials, specifically called calcium silicate-based materials, were introduced into the dental market under sealer and cement forms [5,6,7,8]. Calcium silicate-based cement (named bioceramics) is used as an alternative to the historically used calcium hydroxide. Nowadays there is a wide variety of calcium silicate-based cement in dentistry, and due to their biocompatibility, bioactivity, and biomineralization properties, they have been applied in different clinical procedures including direct and indirect pulp capping, regenerative endodontic treatments, pulpotomy and repairing of perforations [5,6].

However, after the use of a bioceramics material, it is necessary to cover it with a restoration material in order to provide an adequate seal to prevent bacterial penetration and to help it remain in place under dislodging forces [9]. Resin composites are often used as restorative materials, and the quality of the bioceramic–composite bond has a significant impact on the success of the resin composite restoration, and in this sense, the bond strength between the resin composite and calcium silicate-based cement materials is an essential clinical factor for the success of these types of treatments [10,11].

Nowadays, two different adhesion strategies can be used within a clinical scenario [10]. In the total-etch strategy, adhesives are applied after phosphoric acid etching of both enamel and dentin [11]. On the other hand, the self-etch strategy implies the use of self-etch adhesives containing acidic monomers that both etch and prime the dental substrate, and therefore, the application of the phosphoric acid is eliminated in the self-etching technique [12]. Some adhesives can be used in both total-etch or self-etch strategies, constituting what is known as universal adhesives [13].

To date, different studies have been achieved to evaluate the bond strength of resin composites to calcium silicate-based cement using different adhesive systems and strategies [14,15,16]. Considering that the quality of the bond between the calcium silicate-based cement and the composite restoration has been proved to play an important role in the success of the restoration, it is mandatory to establish which adhesive strategy provides the best bond between these materials. Therefore, this systematic review and meta-analysis aimed to assess the effect of different adhesive systems on the bond strength of resin composites to calcium silicate-based cement. The null hypothesis to be tested was that there would be no differences in bond strength to calcium silicate-based cement when using total-etch or self-etch adhesive systems.

## 2. Results and Discussion

**Figure 1:**
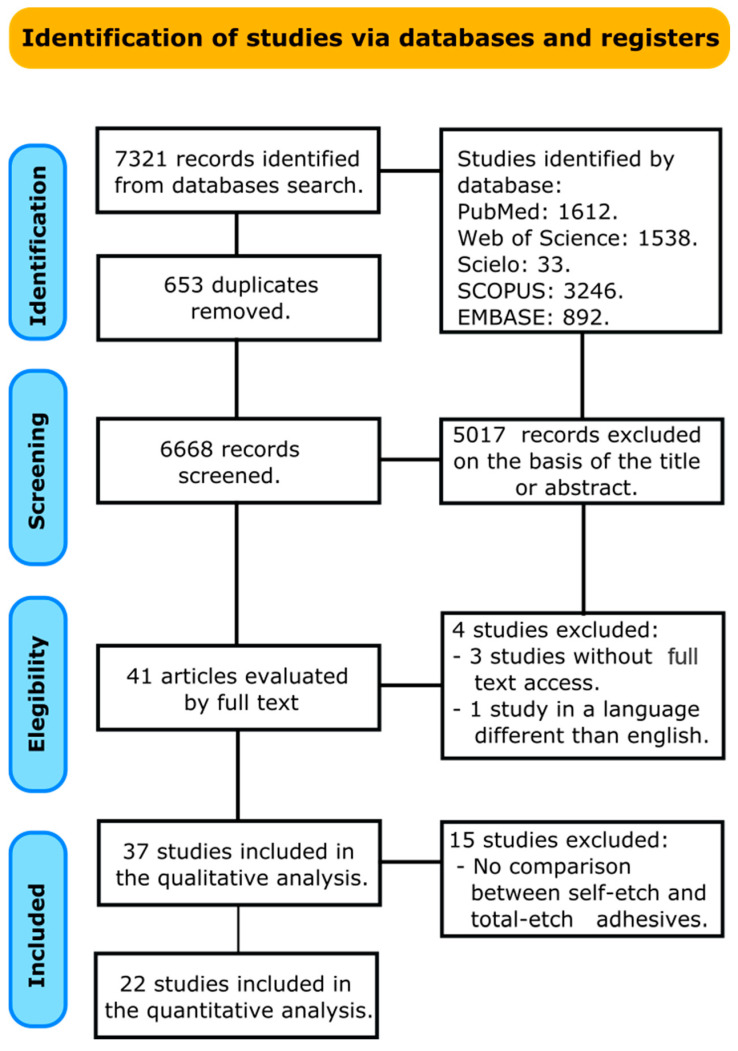
Search flowchart according to the PRISMA Statement.

A total of 7321 papers were recognized in all databases searched. A flowchart that forms the report selection procedure agreeing to the PRISMA Statement is displayed in Figure 1. The literature review rescued 6668 articles for the initial inspection after removing the duplicates. Afterward, 5017 studies were excluded after reviewing the titles and abstracts, leaving 41 articles to be assessed by full-text interpretation. After the full-text assessment, four studies were excluded, three because the access to the full document was not achieved [17,18,19], and one because the manuscript was in a language different than English [20]. A total of thirty-seven manuscripts were included in the qualitative analysis, and from these, fifteen were excluded from the meta-analysis because they did not compare a self-etch adhesive against a total-etch adhesive [10,21,22,23,24,25,26,27,28,29,30,31,32,33,34]. Finally, twenty-two manuscripts were included in the meta-analysis [11,35,36,37,38,39,40,41,42,43,44,45,46,47,48,49,50,51,52,53,54,55].

This review identified three main calcium silicate-based types of cement, including MTA, Biodentine™, and TheraCal LC^®^. The conditions for the setting of the material varied among the studies including using 37 °C with 100% of relative humidity for different periods of time. Restorative materials included composite resins placed using the total-etch or self-etch adhesive technique. Other articles evaluated self-adhesive resin composites and glass ionomer cement. The bond strength test used in the totality of the articles included was the shear bond strength (SBS) test and storing conditions of the samples were in distilled water or saliva for a period of time who ranged from 24 h until 28 days. None of the articles included in this review compared the immediate versus the long-term bond strength (Table 1).

**Figure 2:**
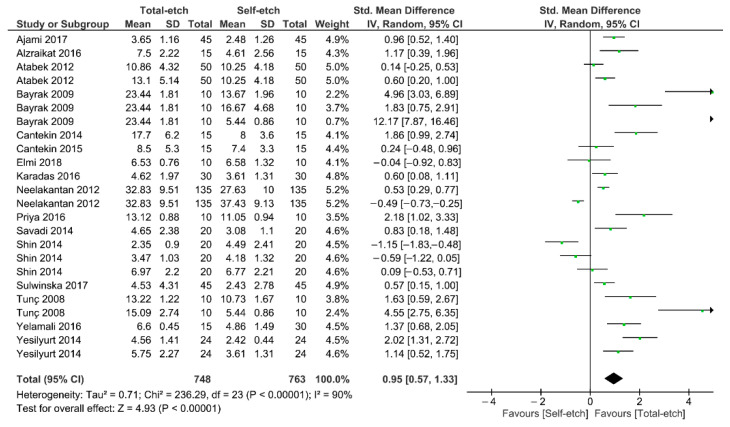
Findings of the meta-analysis of the bond strength of resin-based materials to MTA calcium silicate-based cement. Bond strength was higher when a total-etch adhesive was used (*p* < 0.01).

Figure 2 shows the meta-analysis of the bond strength values of resin composite materials to MTA cement. According to the analysis, the bond strength was enhanced when a total-etch adhesive system was used (*p* < 0.01). A high heterogenicity (90%) was observed.

**Figure 3:**
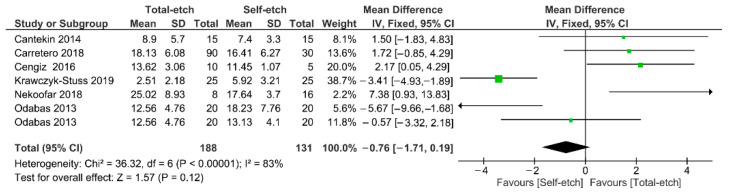
Findings of the meta-analysis of the bond strength of resin-based materials to Biodentine™ calcium silicate-based cement. Bond strength was similar between both strategies (*p* = 0.12).

Figure 3 shows the meta-analysis of the bond strength values of resin composite materials to Biodentine™ cement. According to the analysis, the bond strength was similar between the total-etch and self-etch adhesives (*p* = 0.12). A high heterogenicity (83%) among the studies was observed.

**Figure 4:**
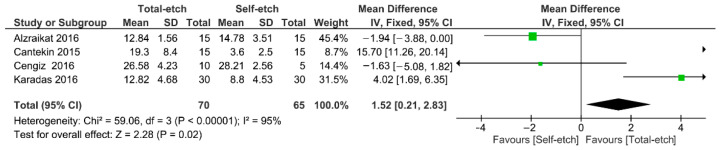
Findings of the meta-analysis of the bond strength of resin-based materials to TheraCal LC^®^ calcium silicate-based cement. Bond strength was higher for the total-etch technique (*p* = 0.02).

Figure 4 shows the meta-analysis of the bond strength values of resin composite materials to TheraCal LC^®^ cement. According to the analysis, the bond strength was higher for the total-etch technique (*p* = 0.02). A high heterogenicity (95%) among the studies was observed.

The risk of bias in the articles studied in this review is recapped in Table 2. Most of the articles included failed to meet the parameters of Single Operator, Blinded Operator, Sample Size Calculation, and Control group. The global analysis showed that the utmost of the articles included were cataloged as high and medium risk of bias.

This systematic review and meta-analysis were directed to assess the effect of different adhesive strategies on the bond strength of resin composites to calcium silicate-based cement. The overall findings revealed that the bond strength of resin composites to MTA and TheraCal LC^®^ cement was favored when a total-etch adhesive was used. However, for Biodentine™ cement the bond strength was similar between both total-etch and self-etch adhesives. Considering this, the null hypothesis tested in this study was partially accepted.

One should bear in mind that MTA are root canal sealers or cement that have been composed of silicate and calcium. Due to favorable outcomes obtained by MTA in addition to its excellent sealing ability, clinical applications, and biocompatibility in endodontic treatment such as root-end filling, pulp capping, apical plug for teeth with open apices, and perforation repair, investigators have been fortified to test materials with comparable promising assets though being less pricey as well as less of the present shortcomings of the unique MTA [56,57]. Higher radiopacity, handling characteristics, prevention of tooth discoloration, and lower setting time of MTA can be modified by new materials with the same composition [58,59,60]. Consequently, calcium silicate-based cement was presented. One calcium silicate-based cement, Biodentine™ (Septodont, Saint-Maur-des-Fossés, France), necessitated quicker setting times, exhibited less discoloration, and presented more satisfactory clinical outcomes than MTA [61,62]. Lately, TheraCal LC^®^, a novel light-cured MTA-filled was introduced in an attempt to enhance mechanical strength, handling properties, and application to tooth substrate. Furthermore, it can be cured immediately using a light curing unit and flow over a surface before it is cured [63]. Nevertheless, in human dental pulp stem cells, TheraCal LC^®^ was stated to be more cytotoxic than Biodentine™ and MTA [64].

In restorative dentistry, resinous materials have increased popularity due to their promising esthetic outcome [55]. Proper bonding between calcium silicate-based cement and resinous materials was considered essential for the ultimate success of dental restorations and the quality of fillings [11]. It should be noted that for evaluating the adhesive properties of restorative materials, the most commonly used test is the bond strength [11]. However, choosing between total-etch or self-etch adhesive systems was considered challenging in such a situation [65].

Agreeing to the meta-analysis, the bond strength values of resin composite materials to MTA and TheraCal LC^®^ cement were favored when a total-etch adhesive system was used (*p* < 0.05). It should be emphasized that a bond strength ranging between 17 to 20 MPa might be needed to challenge contraction forces adequately to generate restoration margins without gap [66]. In this review, SBS was lower using self-etch adhesives and this could be attributed to several factors. Knowing that a chief solvent/oxygen inhibition results through light activation of these materials, a lower degree of conversion of the resin monomer could be observed. In addition, combining hydrophilic and hydrophobic acidic monomers into one bottle might jeopardize the polymerization of the adhesive [67]. Further, the integrally low strength of the adhesive polymer could be responsible for the suboptimal performance of self-etch adhesives [39]. The pH of the self-etch adhesive systems could also play a role, and one of the explanations for the lower bond strength for self-etch adhesives might be the incompatibility between the adhesive and the restorative material [68]. In addition, it should be highlighted that different solvents lead to differences in the bond strength values; however, this variable was not studied in this review. This deduction appears to support the results in this analysis.

The finding showed that the acid-etch technique was deemed crucial for the enhanced bond between MTA and TheraCal LC^®^ cement with resin composites. Acid-etch enhances the wettability of MTA and thus the bond strength with composite resin. Additionally, as a result of an acidic environment, the surface’s porosity increases, causing micro-retention zones during adhesion. It has been demonstrated that after the application of phosphoric acid, the MTA surface is altered, creating gel-like irregular structures and a spindle-shaped surface, which provides a desirable surface for resin materials to bond [46]. Actually, previous research confirmed that the structural and chemical changes within the surface of calcium-based silicate cement occur after 20 s of etching with 37% orthophosphoric acid [68]. Additionally, the high bond strength for TheraCal LC^®^ when using acid-etch could be credited to the presence of dimethacrylate monomer that indorses chemical adhesion between the TheraCal LC^®^ and total-etch adhesive [45]. It can be concluded that a total-etch adhesive would be the substance of choice to reach improved bond strength values when bonding composite resin to MTA and TheraCal LC^®^ cements.

The present analysis revealed that the bond strength values of resin composite materials to Biodentine™ cement were similar between both total-etch and self-etch adhesives (*p* = 0.12). This could be in agreement with a previous study evaluating three adhesive systems and highlighting the variation in the composition of both adhesive systems and resin composites on the outcome of the bond strength to Biodentine™ [49]. One should state that the bond strength between restorative materials and Biodentine™ at several application periods is significant for the longevity of the restorations and their quality [9,41]. Numerous factors could affect this statement such as the low viscosity of adhesive systems which may increase the penetration of adhesive systems into the Biodentine™ cement [69]. Further, the addition of 10-methacryloyloxydecyl dihydrogen phosphate (MDP) into the composition of the adhesive system may facilitate the chemical interaction with the calcium-rich Biodentine™ surface [70,71]. Suitable resin–dentin bonding is usually instantaneously reached, and lessened bonding efficiency arises over time [65]. Low bond strength of aged Biodentine™ could be observed and might be elucidated by the fact that the surface hardness of Biodentine™ increases with time, thus resulting in reduced micro-mechanical retention and shallower etching pattern [48,72]. In summary, the use of both adhesion strategies seem to considerably increase the SBS of resin composites to Biodentine™. 

From this review, the effect of different adhesive systems was analyzed to evaluate their bond strength to calcium silicate-based cement, including MTA, Biodentine™, and TheraCal LC^®^. Considering the results, it seems that the interaction between calcium silicate-based cement and resin-based materials, (i.e., adhesive systems) is more physical, given by the porosities that phosphoric acid creates within the surface of the material [36]. The outcomes of this study should be considered with caution since other calcium silicate-based cement were available in the dental market and not included. Further, the principal restraint of the present review was a lack of assessment of the features and surface morphology of these cement at distinctive setting times and moisture [73], which might have facilitated these findings. In addition, it is recommended that in future reports scanning electron microscopy (SEM) and atomic force microscopy (AFM) evaluation must be carried out in an attempt to supplementary elucidate the explanations for increases and/or decreases in SBS of these cements using different strategies. Further, most of the articles included were categorized as having high or medium risk of bias, and, consequently, superior investigational designs must be directed in order to obtain a higher degree of evidence. Moreover, clinical studies were needed since providing the best bond between calcium silicate materials and resin composites was scarce.

## 3. Conclusions

The findings of the review suggest that the bond strength of resin-based materials to both MTA and TheraCal LC^®^ cement could be enhanced by the use of the total-etch technique. On the other hand, when bonding to Biodentine™ the use of self-etch or total-etch strategies displayed promising results. Given the lack of evidence related to the chemical interaction of self-etch adhesive materials with the bioceramics, if self-etch adhesives are used for bonding resin-based restorations to calcium silicate-based cement, a pretreatment with phosphoric acid could be recommended.

## 4. Materials and Methods

The present study was conducted adhering to the guidance of PRISMA [73]. The following PICOS framework was used: population, calcium silicate-based cement; intervention, use of self-etch adhesive systems; control, use of total-etch adhesive systems; outcome, bond strength; study design, in vitro studies. The research interrogation was: what is the adhesive strategy that provides the highest bond strength between calcium silicate-based cement and resin-based materials?

### 4.1. Literature Search

The systematic search was conducted independently by two authors (AAO and CECS) up to 18 February 2021, without date restriction, among five electronic databases (PubMed, ISI Web of Science, SciELO, Scopus, and Embase). The keywords and search strategy used in PubMed and adapted to the other data search engines are listed in Table 3. The reviewers also performed a manual search of reference lists of the included articles for supplementary literature. All articles located in the databases were imported into the Mendeley Desktop 1.17.11 software to remove duplicates.

### 4.2. Study Selection

Titles and abstracts were initially screened by two reviewers (LH and RB) in order to identify studies that potentially met the following eligibility criteria: (1) in vitro studies reporting the bond strength of total-etch and self-etch adhesive systems to calcium silicate-based cement; (2) studies including mean and standard deviation (SD) data in MPa on shear, microshear, tensile, and micro-tensile bond tests; (4) studies published in English. Case reports, case series, pilot studies, and reviews were excluded from the initial review. Full copies of all of the possibly applicable articles were inspected. If after reading the title and abstract, it was not possible to make a clear judgment, the article was designated for full analysis. The full-text manuscripts were evaluated individually in duplicate by two investigators. Any inconsistency or variation concerning the suitability of the comprised manuscripts was determined through consultation with a third reviewer, a senior experienced researcher (CECS). Only manuscripts that encountered the appropriateness criteria were integrated for review.

### 4.3. Data Extraction

Data of interest from the comprised articles were inserted into standardized worksheets using Microsoft Office Excel 2021 software (Microsoft Corporation, Redmond, WA, USA). These data included the year of publication, author, bioceramic used as substrate, setting conditions (time and temperature), adhesive system used, outcomes evaluated (mean, SD, *n*), and storing conditions. If any information was missing the corresponding author of the article was contacted to supply the exact data. If a response was not obtained within 2 weeks of the first contact, the missing information was not comprised.

### 4.4. Quality Assessment

The methodological quality of the included studies was evaluated by two reviewers (AAO and LH) considering the parameters of previous systematic reviews [74,75]. The risk of bias in each article was assessed according to the description of the following parameters: sample randomization; single-operator protocol implementation; blinding of the operator; the presence of a control group; standardization of the sample preparation; use of all materials according to the manufacturer’s instructions; and description of the sample size calculation. If the parameter was described within the study, the study received a “YES”. In the case of omitted data, the factor received a “NO”. The risk of bias was classified according to the sum of “YES” answers received: 1 or 2 indicated a high bias, 3 to 5 medium, and 6 or 7 indicated a low risk of bias.

### 4.5. Statistical Analysis

The meta-analyses were accomplished using a software program (Review Manager version 5.3.5; The Cochrane Collaboration, Copenhagen, Denmark). A random-effect model was used to carry out the different analyses by comparing the standardized mean difference of the bond strength values using the total-etch or self-etch adhesive systems. Bond strength comparisons were completed bearing in mind the calcium silicate-based cement (Mineral Trioxide Aggregate, Biodentine™, or TheraCal LC^®^). A *p*-value < 0.05 was contemplated statistically significant. The heterogeneity was calculated using the Cochran Q test and the inconsistency I^2^ test.

## Figures and Tables

**Table 1:** Demographic data.

Author	Bioceramic Used as Substrate	Bioceramic Setting Conditions	Bonding Systems Used	Composite Resin	Storing Conditions	Bond Strength Test	Secondary Outcome
Ajami, 2013	CEM (Bionique Dent; Tehran, Iran). NAMTA (produced by dr. Lotfi in Tabriz Dental Faculty, Tabriz, Iran). MTA (Dentsply; Tulsa Dental, OK, USA)	37 °C with 100% humidity relative for 24 h.	Total-etch adhesive (Adper TM Single Bond; 3M ESPE, St. Paul, MN, USA)	Filtek TM Z250 (3M ESPE Dental Products, St. Paul, MN, USA)	100% relative humidity at 37 °C for 24 h.	Shear bond strength (SBS)	Fracture analysis
Ajami, 2017	MTA	37 °C in 100% humidity for 2.45 h and 3 days.	Total-etch adhesive (Adper Single Bond II™; 3M ESPE, St Paul, MN, USA) Self-etch adhesive (Beautibond Sho-fu Inc., Kyoto, Japan)	Valux Plus composite resin (3M ESPE, St Paul, MN, USA)	37 °C in 100% humidity for 24 h.	SBS	-
Altunsoy, 2015	MTA (Angelus, Londrina, PR, Brazil) Biodentine™ (Septodent, Saint-Maur-des-Fosses Cedex, France) CEM (Yektazist Dandan, Tehran, Iran)	100% humidity at 37 °C for 72 h.	Self-adhesive restorative (Vertise Flow (Kerr, Orange, CA, USA) Etch-and-rinse adhesive (Futurabond DC; Voco GmbH, Cuxhaven, Germany)	X-tra base (Voco GmbH, Cuxhaven, Germany)	100% relative humidity at 37 °C for 24 h.	SBS	Fracture surface analysis
Alzraikat, 2016	ProRoot Mineral Trioxide Aggregates (Dentsply Tulsa Dental, Tulsa, OK, USA) TheraCalTM LC (BISCO Dental Products, Schaumburg, IL, USA) GC Fuji IX GP (GC, Tokyo, Japan)	100% humidity and 37 °C for 48 h Polymerized with a light curing for 20 s 37 °C in 100% humidity for 24 h.	Total-etch adhesive (AdperTM Single bond 2; 3M ESPE, St. Paul, MN, USA) Self-etch adhesive (Xeno V; Dentsply, Tulsa, OK, USA)	Filtek™ Z250 (3M ESPE, St. Paul, MN, USA)	100% relative humidity at 37 °C for 24 h.	SBS	Fracture surface analysis
Atabek, 2012	WMTA	37 °C with 100% humidity for 4, 24, 48, 72, and 96 h.	Total-etch adhesive (One-Step Plus and All-Bond 3; Bisco Inc, Schaumburg, IL, USA) Self-etch adhesive (All-Bond SE; Bisco, Inc, Schaumburg, IL)	Composite Aelite (Bisco Inc, Schaumburg, IL, USA)	100% relative humidity at 37 °C for 24 h.	SBS	Fracture Analysis
Bayrak, 2009	White MTA (Dentsply, Tulsa, OK, USA)	37 °C with 100% humidity for 48 h.	Total-etch adhesive (Prime & Bond NT; Caulk/Dentsply International Inc., Milford, DE, USA) Self-etch adhesive (AdheSE; Ivoclar Vivadent, Schaan, Liechtenstein. Xeno III; Dentsply DeTrey, Konstanz, Germany. Adper Prompt L-Pop; 3M ESPE, St. Paul, MN, USA)	Dyract AP (Dentsply DeTrey, Konstanz, Germany)	100% relative humidity at 37 °C for 24 h.	SBS	Fracture analysis
Cantekin, 2014	Biodentine™ (Septodont, Saint Maur des Fosses, France) ProRoot MTA (Dentsply, Tulsa, OK, USA)	37 °C in 100% humidity for 15 min and 96 h for setting.	Total-etch adhesive (One-Step Plus; Bisco Inc, Schamburg, IL, USA) Self-etch adhesive (Filtek Silorane Bond; 3M ESPE, St. Paul, MN, USA)	Aelite All Purpose Body (Bisco Inc, Schamburg, IL, USA) Filtek Silorane (3M ESPE, St. Paul, MN, USA)	37 °C in 100% humidity for 24 h.	SBS	-
Cantekin, 2015	TheraCal (Bisco, Inc., Schamburg, IL, USA). White MTA (ProRoot MTA, Dentsply, Tulsa, OK, USA)	light-cured with an LED cure unit 100% humidity at 37 °C for 96 h.	Total-etch adhesive (One-Step Plus; Bisco Inc, Schamburg, IL, USA) Self-etch adhesive (Silorane Bond; 3M ESPE, St. Paul, MN, USA)	AELITE (Bisco, Inc. Schamburg, IL, USA) Filtek Silorane (3M ESPE, St. Paul, MN, USA) GC Fuji IX (GC, Tokyo, Japan)	100% relative humidity at 37 °C for 24 h.	SBS	Fracture surface analysis
Carretero, 2018	Biodentine™ (Septodont, Saint Maur des Fosses, France)	37 °C and 100% humidity for 12 min and 24 h.	Total-etch adhesive (Optibond FL; Kerr Corp, Orange, CA, USA. Solobond M; Voco GmbH, Cuxhaven, Germany) Universal adhesive (Scotchbond Universal; 3M ESPE, St. Paul, MN, USA)	Grandio^®^ (VOCO GmbH, Cuxhaven, Germany)	37 °C in 100% humidity for 24 h.	SBS	Fracture surface analysis
Cengiz, 2016	TheraCal LC (BISCO Dental Products, Schaumburg, IL, USA) Biodentine™ (Septodont, Saint Maur des Fosses, France)	Light cured for 20 s 12 min.	Universal Adhesive (Single bond Universal, 3M ESPE; St Paul, MN, USA) Total-etch adhesive (Prime&Bond NT; Dentsply DeTrey; Konstanz, Germany) Self-etch adhesive system (Clearfil SE Bond; Kuraray Noritake; Tokyo, Japan) Self-adhering flowable composite (Vertise Flow; Kerr, Orange, CA, USA)	Filtek Bulk Fill Posterior Restorative (3M ESPE; St Paul, MN, USA) Filtek Z250 composite resin (3M ESPE; St Paul, MN, USA)	100% relative humidity at 37 °C for 24 h.	SBS	Fracture surface analysis
Colak, 2016	Biodentine™ (Septodont, Saint Maur des Fosses, France)	37 °C in 100% humidity for 9 min, and 48 h.	Self-etch adhesive (Clearfil S3 Bond; Kuraray Medical, Osaka, Japan. Adper Prompt L-Pop; 3M/ESPE, St. Paul, MN, USA) Total Etch adhesive (Prime Bond N&T; Dentsply, Konstanz, Germany)	Filtek Z250, (3M/ESPE, St. Paul, MN, USA)	37 °C in 100% humidity for 48 h.	SBS	Fracture surface analysis
Doozaneh, 2017	RootMTA (Tabriz, Iran) CEM (Bionique Dent, Tehran, Iran)	37 °C in 100% humidity for 72 h.	Total-etch adhesive (OptiBond all-in-one; Kerr Dental, Orange CA, USA) Self-adhering composite (Vertise Flow; Kerr, Orange, CA, USA)	Vertise Flow (Kerr, Orange, CA, USA) RMGI (Fuji II Lc; GC Corp.; Tokyo, Japan)	37 °C in 100% humidity for 24 h.	SBS	Fracture surface analysis
Elmi, 2018	CEM cement (Yektazist Dandan, Tehran, Iran)	37 °C and 100% humidity for 48 h.	Total-etch adhesive (Single bond 2;3M, St. Paul, MN, USA) Universal adhesive (Single Bond Universal; 3M, St. Paul, MN, USA)	Z250 (3M ESPE, St. Paul, MN, USA)	37 °C in 100% humidity for 24 h.	SBS	Fracture surface analysis
Ha, 2019	GC Fuji IX GP Capsule A2 (GC CORPORATION, Tokyo, Japan) Biodentine™ (Septodont, Saint Maur des Fosses, France)	37 °C and 100% humidity for 12 min, 72 h and 2 weeks.	Total-etch adhesive (Optibond FL; Kerr Dental, Orange, USA)	G-aenial Universal Flow (GC CORPORATION, Tokyo, Japan)	37 °C in 100% humidity for 90 min.	SBS	Fracture surface analysis
Hursh, 2019	White ProRoot mineral trioxide aggregate (MTA) (Dentsply Tulsa Dental, Tulsa, OK) Biodentine™ (Septodont, Saint Maur des Fosses, France) EndoSequence Root Repair Material Fast Set Putty (Brasseler USA, Savannah, GA, USA) NeoMTA (Avalon Biomed Inc., Houston, TX, USA)	37 °C and 100% humidity for the manufacturer’s full.	Self-etch adhesive (Clearfil SE Bond; Kuraray America, Inc., New York, NY, USA)	Clearfil DC Core Plus (Kuraray America, Inc.)	37 °C in 100% humidity for 7 days.	SBS	Fracture surface analysis
Karadas, 2016	TheraCal LC^®^ (Bisco; Schaumburg, IL, USA) MTA-AMTA (Angelus Industria; Londrina, PR, Brasil)	Light-curing for 20 s 37 °C in 100% humidity for 72 h.	Total-etch adhesive (Adper Scotchbond Multipurpose 3M; ESPE, Seefeld, Germany) Self-etch adhesive (Clearfil SE Bond; Kuraray America, Inc., New York, NY, USA. Clearfil Protect Bond; Kuraray America, Inc., New York, NY, USA. Clearfil S3 Bond; Kuraray America, Inc., New York, NY, USA. OptiBond All In One; Kerr Dental, Orange, USA. G-aenial Bond; GC America, USA)	Filtek Z250 (3M ESPE; Saint Paul, MN, USA)	37 °C in 100% humidity for 24 h.	SBS	Fracture surface analysis
Krawczyk-Stuss, 2019	Biodentine™ (Septodent, Saint-Maur-des-Fosses Cedex, France)	12 min in 0.9% NaCl.	Total-etch adhesive (Adper Single Bond; 3M ESPE, Seefeld, Germany) Self-etch adhesive (Clearfil S3 Bond Plus; Kuraray Medical, Kurashiki, Japan)	Filtek Z250 (3M ESPE, St. Paul, MN, USA)	-	SBS	-
Murat, 2018	Biodentine™ (Septodent, Saint-Maur-des-Fosses Cedex, France)	37 °C with 100% humidity during for 12 min, 24 h and 2 weeks	Universal Adhesive (Single Bond Universal; 3M Espe, St. Paul, MN, USA)	Tetric EvoCeram Bulk Fill (Ivoclar Vivadent, Schaan, Lichtenstein) Filtek Bulk Flow (3M ESPE St Paul, MN, USA) Beautifil Bulk (SHOFU Kyoto, Japan Filtek Bulk Fill Posterior (3M ESPE St Paul, MN, USA) SDR (DENTSPLY Caulk, Milford, DE, USA)	37 °C in 100% humidity for 24 h	SBS	Fracture surface analysis SEM
Mustafa, 2020	Biodentine™ (Septodent, Saint-Maur-des-Fosses Cedex, France)	37 °C in artificial saliva for 12 min, 14, and 28 days.	Total-etch adhesive (AdperTM Single; 3M Oral Care, St. Paul, MN, USA)	Tetric EvoCeram (Ivoclar Vivadent AG, Schaan, Liechtenstein).	37 °C in artificial saliva for 30 days.	SBS	Fracture surface analysis
Nagi, 2020	MTA Angelus (Angelus, Londrina, PR, Brazil) EndoSequence BC RRM-Fast Set PuttyTM (ERRM) (Brasseler, USA)	37 °C with 100% humidity during for 72 h.	Universal adhesive (Single bond universal; 3M ESPE. St. Paul, MN, USA)	Filtek Z250 (3M ESPE. St. Paul, MN, USA)	37 °C in 100% humidity for 24 h.	SBS	Failure analysis
Neelakantan, 2012	WMTA	Immediate, 45 min and 24 h.	Total-etch adhesive (Prime & Bond NT; Dentsply Caulk, Milford, Del.) Self-etch adhesive (AdheSE; Ivoclar Vivadent, Schaan, Liechtenstein. Clearfil S3 Bond; Kuraray Dental, Tokyo)	Filtek Z350 (3M ESPE, St. Paul, MN, USA.)	100% relative humidity at 37 °C for 24 h.	SBS	Fracture analysis
Nekoofar, 2018	Biodentine™ (Septodont, Saint Maur des Fosses, France)	37 °C and 100% humidity for 12 min, one week, and one month.	Total-etch adhesive system (AdperTM Single Bond 2; 3M/ESPE, St.Paul, MN, USA) Self-etch adhesive (Clearfil SE Bond; Kuraray, Okayama, Japan) Universal adhesive (All-Bond Universal, Bisco, Schaumburg, IL, USA)	Filtek Z350 XT (3M/ESPE, St. Paul, MN, USA)	37 °C in 100% humidity for 2 days.	SBS	-
Odabas, 2013	Biodentine™ (Septodont, Saint-Maur-des-Fosses Cedex, France)	37 °C with 100% humidity for 12 min and 24 h.	Total-etch adhesive (Prime & Bond NT; Caulk/Dentsply International Inc., Milford, DE, USA) Self-etch adhesive system (Clearfil SE Bond; Kuraray Noritake Dental Inc, Okayama, Japan. Clearfil S3 Bond; Kuraray Noritake Dental Inc, Okayama, Japan)	Clearfil Mejesty, Kuraray (Noritake Dental Inc, Okayama, Japan)	100% relative humidity at 37 °C for 24 h.	SBS	Fracture Analysis
Palma, 2018	ProRoot MTA (Dentsply Tulsa Dental) Biodentine™ (Septodont, Saint Maur des Fosses, France)	37 °C in 100% humidity for 12 min and 7 days.	Universal Adhesive (Prime&Bond Active™; Dentsply Detrey GmgH, Konstanz, Germany)	SDR (Dentsply Detrey GmgH, Konstanz, Germany)	37 °C in 100% humidity for 48 h.	SBS	Fracture surface analysis
Palma, 2020	Biodentine™ (Septodont, Saint-Maur-des-Fossés Cedex, France) TotalFill BC RRM (Putty FKG, La Chaux-de-Fonds, Switzerland) PCM (Coltène/Whaledent, Altstätten, Switzerland)	37 °C and 100% relative humidity for 12 min and 7 days.	Self-etch adhesive (Clearfil SE Bond; Kuraray Medical, Okayama, Japan)	SDR Bulk fill flowable composite (Dentsply DeTrey GmbH, Konstanz, Germany)	37 °C with 100% humidity for 48 h.	SBS	Fracture surface analysis
Priya, 2016	White MTA (ANGELUS)	37 °C with 100% humidity in an incubator for 24 h.	Total-etch adhesive (Adper Single Bond 2; 3M ESPE, St.Paul, MN, USA) Self-etch adhesive (Xeno V; Dentsply Detrey GmgH, Konstanz, Germany) Self-adhering flowable composite (Vertise flow; Kerr)	Filtek Z250 Z250 (3M ESPE; Saint Paul, MN, USA) Ceram X mono composite (Dentsply Detrey GmgH, Konstanz, Germany).	37 °C with 100% humidity for 24 h.	SBS	-
Raina, 2020	Dycal (Dentsply Caulk, Milford, DE, USA) MTA Plus (Prevest Denpro, Jammu, India) Biodentine™ (Septodont, Saint-Maur-des-Fossés, France) TheraCal (Bisco, Inc., Schaumburg, IL, USA)	37 °C and 100% relative humidity for 72 h.	Self-etch adhesive (OptiBond; Kerr Dental, Orange, USA) Self-adhering flowable composite Dyad Flow (Kerr Dental, Orange, USA)	SDR (Dentsply DeTrey GmbH, Konstanz, Germany)	37 °C and 100% relative humidity for 24 h.	SBS	Scanning electron microscopy (SEM).
Samimi, 2018	ProRoot MTA (Dentsply, Tulsa, OK, USA)	37 °C in 100% humidity for 72 h.	Total-etch adhesive (OptiBond Solo Plus; Kerr, Karlsruhe, Germany)	Point 4 (3M ESPE, St. Louis, MN, USA)	37 °C in 100% humidity for 24 h.	SBS	SEM analysis
Savadi, 2014	Calcium Enriched Mixture (BioniqueDent Tehran, Iran). ProRoot MTA (Dentsply Tulsa Dental, Johnson City, TN, USA)	37 °C with 100% humidity for 50 min. 37 °C with 100% humidity for 48 h.	Total-etch adhesive (Single Bond; 3M ESPE, St. Paul, MN, USA) Self-etch adhesive (Clearfil SE Bond; Kuraray Medical Inc., Tokyo, Japan)	Gradia Direct (GC Corporation, Tokyo, Japan)	100% relative humidity at 37 °C for 24 h.	SBS	-
Schmidt, 2017	MTA-Angelus Angelus (Londrina, Brazil) Biodentine™ (Septodont St. Maur-des-Fossés, France)	3 min 15 min 2 days.	Self-etch adhesive (Futurabond NR; VOCO, Cuxhaven, Germany) Self-adhering flowable composite Dyad Flow (Kerr Dental, Orange, USA)	Ionoseal (VOCO, Cuxhaven, Germany) Grandio Flow (VOCO, Cuxhaven, Germany)	37 °C in 100% humidity for 28 days.	SBS	Fracture surface analysis
Shin, 2014	ProRoot MTA (WMTA) (Dentsply, Tulsa, OK) MTA Angelus (AMTA) (Angelus, Londrina, PR, Brazil) Endocem MTA (EMTA) (Maruchi, Wonju, Korea)	100% humidity at 37 °C for a week.	Total-etch adhesive (Scotchbond Multipurpose; 3M ESPE, St Paul, MN, USA. Single Bond 2; 3M ESPE, St Paul, MN, USA.). Self-etch adhesive (Clearfil SE BOND; Kuraray, Osaka, Japan. AdheSE One F; Ivoclar Vivadent, Schaan, Liechtenstein)	Filtek Flow (3M ESPE, St Paul, MN, USA)	100% relative humidity at 37 °C for 24 h.	SBS	Scanning Electron Microscopic Analysis
Sulwinska, 2017	Pro Root MTA (Dentsply Tulsa Dental Specialities, Johnston City, USA)	Directly after the application 24 h 72 h.	Universal adhesive (Single Bond Universal; 3M ESPE, St. Paul, MN, USA)	Filtek Ultimate (3M ESPE, St. Paul, MN, USA)	37 °C with > 95% relative humidity.	SBS	Fracture surface analysis
Tulumbaci, 2017	Pro Root MTA (Dentsply Tulsa Dental Specialities, Johnston City, USA) Biodentine™ Septodont (St. Maur-des-Fossés, France)	37 °C in 100% humidity for 72 h.	Total-etch adhesive (Prime and Bond NT; Dentsply, Tulsa, OK, USA )	Filtek Z250 (3M ESPE, USA) Compomer Dyract XP (LD Caulk/ Dentsply, Tulsa, OK, USA)	37 °C in 100% humidity for 24 h.	SBS	Fracture surface analysis
Tunc, 2008	WMTA (Dentsply, Tulsa Dental)	37 °C with 100% humidity for 48 h.	Total-etch adhesive (3M/ESPE, St Paul, MN, USA) Self-etch adhesive (Prompt L-Pop; 3M Dental Products, St Paul, MN, USA)	Filtrek Z250 (3M/ESPE, St Paul, MN, USA) Dyract AP (Dentsply DeTrey, Konstanz, Germany)	100% relative humidity at 37 °C for 24 h.	SBS	-
Yelamali, 2016	White MTA (Angelus, Londrina, Brazil)	37 °C with 100% humidity in an incubator for 24 h.	Total-etch adhesive (Adper Single Bond 2; 3M/ESPE, St. Paul, MN, USA) Self-etch adhesive (Clearfil SE Bond; Kuraray, Medical Inc. G Bond; GC Corporation, Tokyo, Japan)	Filtek Z350 (3M ESPE, St Paul, MN, USA)	100% relative humidity at 37 °C for 24 h in an incubator.	SBS	-
Yesilyurt, 2014	BioAggregate (BA, Innovative BioCeramix, Vancouver, Canada)	100% humidity at 37 °C for 24 and 72 h.	Self-etch adhesive (Clearfil SE Bond; Kuraray Noritake Dental, Tokyo, Japan) Total-etch adhesive (Scotch Bond Multi-Purpose Adhesive System; 3M ESPE, St. Paul, MN, USA) Self-adhering flowable composite Dyad Flow (Kerr Dental, Orange, USA)	Ultimate Flow (UF; Kerr, Orange, CA, USA)	100% relative humidity at 37 °C for 24 h.	SBS	Fracture surface analysis
Zarean, 2019	Mineral trioxide aggregate (ProRoot^®^ MTA; Dentsply Sirona Inc., New York, NY, USA) CEM cement (Yektazist Dandan, Tehran, Iran) Biodentine™ (Septodont, Saint- Maur-des-Fossés, France)	37 °C and 100% relative humidity for 24 h.	Total-etch adhesive (Solobond M; VOCO GmbH, Cuxhaven, Germany)	Grandio Flow composite resin (VOCO GmbH, Cuxhaven, Germany)	37 °C and 100% relative humidity for 24 h.	SBS	Fracture surface analysis

**Table 2:** Risk of bias assessment. (Red Code = High risk; Black code = Medium risk).

Author	Sample Randomization	Single Operator	Blinded Operator	Sample Dimensions	Sample Size Calculation	Control Group (without Pretreatment)	Manufacturer Instructions	Risk of Bias
Ajami, 2013	NO	NO	NO	YES	NO	NO	YES	High
Ajami, 2017	YES	NO	NO	YES	YES	NO	YES	Medium
Altunsoy, 2015	NO	NO	NO	YES	NO	NO	YES	High
Alzraikat, 2016	NO	NO	NO	YES	NO	NO	YES	High
Atabek, 2012	YES	NO	NO	YES	NO	YES	YES	Medium
Bayrak, 2009	NO	NO	NO	YES	NO	NO	YES	High
Buldur, 2018	YES	NO	NO	YES	NO	NO	YES	Medium
Cantekin, 2014	YES	YES	NO	YES	NO	NO	YES	Medium
Cantekin, 2015	YES	NO	NO	YES	NO	NO	YES	Medium
Carretero, 2018	NO	NO	NO	YES	NO	NO	YES	High
Cengiz, 2016	YES	NO	NO	YES	NO	NO	YES	Medium
Colak, 2016	YES	YES	NO	YES	NO	NO	YES	Medium
Doozaneh, 2017	NO	NO	NO	YES	NO	NO	YES	High
Elmi, 2018	NO	NO	NO	YES	NO	NO	YES	High
Ha, 2019	NO	NO	NO	YES	NO	NO	YES	High
Hursh, 2019	NO	NO	NO	YES	NO	NO	YES	High
Karadas, 2016	NO	NO	NO	YES	NO	NO	YES	High
Krawczyk-Stuss, 2019	NO	YES	NO	YES	NO	YES	YES	Medium
Mustafa, 2020	YES	NO	NO	YES	YES	NO	YES	Medium
Nagi, 2020	NO	NO	NO	YES	NO	NO	YES	High
Neelakantan, 2012	YES	YES	YES	YES	NO	NO	YES	Medium
Nekoofar, 2018	YES	NO	NO	YES	NO	YES	YES	Medium
Odabas, 2013	YES	NO	NO	YES	NO	YES	YES	Medium
Palma, 2018	YES	NO	NO	YES	NO	NO	YES	Medium
Palma, 2020	YES	NO	NO	YES	YES	NO	YES	Medium
Priya, 2016	NO	NO	NO	YES	NO	NO	YES	High
Raina, 2020	NO	NO	NO	YES	NO	NO	YES	High
Samimi, 2018	NO	NO	NO	YES	NO	YES	YES	Medium
Savadi, 2014	NO	NO	NO	YES	NO	NO	YES	High
Schmidt, 2017	NO	NO	NO	YES	NO	NO	YES	High
Shin, 2014	YES	NO	NO	YES	NO	NO	YES	Medium
Sulwinska, 2017	NO	YES	NO	YES	NO	YES	YES	Medium
Tulumbaci, 2017	NO	NO	NO	YES	NO	NO	YES	High
Tunc 2008	NO	NO	NO	YES	NO	NO	YES	High
Yelamali, 2016	YES	NO	NO	YES	NO	NO	YES	Medium
Yesilyurt, 2014	NO	NO	NO	YES	NO	YES	YES	Medium
Zarean, 2019	NO	NO	NO	YES	NO	NO	YES	High
Ünal, 2018	NO	NO	NO	YES	NO	NO	YES	High

**Table 3:** Keywords used in search strategy.

#1	Calcium silicate-based cements, calcium-silicate cement, tri-calcium based cements, mineral trioxide aggregate (MTA), ProRoot MTA, MTA-Angelus, Biodentine, TheraCal LC, silicate cement, tricalcium silicatecalcium based
#2	Universal bonding agent, composite, restorative materials, resin composite, universal adhesive, flowable composite, adhesive systems, total-etch adhesive, self-etch adhesive
#3	Bond, bonding, shear bond strength, bonding performance, bonding effectiveness, bonding properties, microtensile bond strength, microshear bond strength, shear bond strength test

## Data Availability

The data that support the findings of this study are available from the corresponding author upon reasonable request.
